# Female Medical Colleges and Female Doctors

**Published:** 1857-11

**Authors:** 


FEMALE MEDICAL COLLEGES AND FEMALE DOCTORS.—Truly was it said by J. R. Tyson, LL.D.,* in reference to the foundation of Female Medical Colleges: "No country but ours could have given them existence. The prejudices of society would not permit them to flourish elsewhere." The remark of the speaker, like the utterance of the Delphic oracle, admits of a double construction: a compliment or a satire. Our own view may be inferred from the manner in which we propose to treat the subject. No country but Dahomey, a kingdom in Western Africa, could have given existence to regiments of female soldiers,—sable amazons, who constitute the body-guards of the king, and are his most trusted troops. The prejudices of society do not yet allow of such a military organization flourishing in either Europe or America; but with the advances made in favor of woman's rights, there is no saying that we may not live to hear of a corps of American mounted amazons scouring the great plains in pursuit of the Camanches and other Indians. In such an event, there will of course be female military surgeons, who have been educated in Female Medical Colleges by Female Professors; and thus feminality will have shown itself triumphant both in arts and arms. What remains but to give women seats in the Senate and in the Representatives Hall; and as any limitation or restriction must grate on their feelings of independence, when once they have begun to run a rival career with men, they cannot be refused a seat on the Bench, or the privilege of expounding and dispensing, as they had before that of making the laws of the land. Shakspeare represents Portia, in the "Merchant of Venice," disguised in man's attire, and pleading as a doctor of laws for Antonio. Our female lawyers and judges need not don man's attire; their sexuality will be disguised enough under the changed features, voice, and manners of their new callings. Mr. Tyson's good nature made him orator, for the nonce, of the Pennsylvania Female College; but his better taste would not permit him to become its direct advocate. On the contrary, he intimates very clearly his opinions of the relative share of the employments of the two sexes, and deprecates the idea of women going out into the world, to struggle with men in the arena of politics or the marts of trade. "Man has the qualities which enable him to breast the storm, while woman's genius enables her to embellish the retreats which form its sheltered coverts." Elsewhere he had said, "From her nature and organization she proves herself to be

——— "'Born to dignify *retreat*,
*Unseen* to flourish and *unknown* be great.'"

He instances, in a letter preceding the address, "departments for which women are well, nay, *peculiarly* qualified." "For example, several of the fine arts, especially *engraving*, *the arts of design*, *watchmaking*, and a variety of kindred employments, are all essentially *feminine*."* Nothing is said in

* Address at the First Annual Commencement of the Pennsylvania Female College at Harrisburg, 1854.

* The italics are all by the author of the address.

advocacy or explanation of females practising medicine; but much is said about their station and duties, as recorded both in sacred and profane history.

Doctor Fussell, Professor of Obstetrics and Diseases of Women and Children in the Female Medical College of Pennsylvania, delivered a valedictory address to the graduating class, Feb. 28th, 1857. From it we select the following passage: "The admission of women into the profession of medicine is not yet fully acquiesced in." The lecturer himself, while enumerating "the qualities necessary to form the character of the good doctor," leaves a strong doubt on the minds of his readers, of the ability of females to come up to one of the requirements which he lays down. It is the possession of "strong, vigorous health. The labors, hardships, and exposures to which the doctor is subject, require the power of great endurance." "The doctor's duties lead him through torrid heat and furiously driving snows: darkness around, and the crashing of the thunderbolt overhead, must not deter him. The bitter blasts and the pelting rain must be encountered." At this particular point in his address, Dr. Fussell ought to have proved to his auditors that woman, with her peculiarities of organization—her uterine system, with its important and delicate functions, its frequent derangements and serious diseases, and the number and complication of sympathetic disorders in other organs consequent on irritation in it—can encounter the exposures to atmospherical extremes, and the toil and hardships incident to professional life, under which so many of the other sex break down. How will she be able to visit her patients and attend to night-calls, as well as those by day, when she is suffering from disordered menstruation, dysmenorrhœa, for example, or from the incidental but frequent ailments attending gestation, or is unavoidably confined to her room during parturition, and every now and then detained at home by an inflamed mamma, or other, though less painful, incidents of lactation? Woman is liable to a train of organic and functional diseases peculiar to her sex, in addition to the long list common to her and to man. She encounters of necessity, many impediments and interruptions in the discharge of professional duties to which man is not subjected; and to this extent she is less fitted than he is for a medical life. Considering the nature of their avocations, when once enlisted in this way, and the trials of constitution to which they will be subjected, we are fully of opinion that women could better imitate the sable amazons of Dahomey, by forming themselves into regiments of mounted dragoons, than engage in the practice of medicine.

So far, reference has only been made to the strain on woman's physical powers: that on her mental ones is still greater and more trying. With her greater sensibility than man's, her brain must be in a state of continual excitement from the scenes which she will be compelled to witness; and the effects on a mobile organization and nervous temperament, in consequence, can hardly fail to be of a sinister nature, and to prevent her from displaying that cheerfulness which is one of her attributes, and which is laid down by Dr. Fussell as a requirement "necessary to form the character of a good doctor." There can be no dispute as to the necessity of another one designated by him, viz., common sense. Of the possession of this quality by the sex, we find hourly proof in her legitimate domestic and social relations. Some cynics may be heard to say, that the very fact of a woman's intending to practise medicine is a proof that she and common sense are no longer in partnership. It will be alleged, also, by those who hold this opinion, that, in one respect, a woman determined to become a practising physician must have undergone such a seasoning, an induration, as it were, of her feminine sensibilities, as to protect her from suffering any great shock to her feelings. After this process, she might be compared to the "Mock Doctor" in the English play, *Le Medecin Malgré Lui* in the French, who, at first, a simple woodman, binding up faggots, is lighted on, by the mistake of a party sent in quest of a physician, living in retirement in the forest. Our woodman at first protests that he is ignorant of medicine; but he is forced, by dint of a good cudgelling, to admit that he is a doctor, and is ready to obey their bidding. The medical induction is hardly more severe in the one case than in the other; but with this difference, that it is self-imposed by the female doctor; and that in place of throwing off a homely peasant's garb, she parts with the veil of modesty, and unrobes herself of the delicacy which is at once her charm and protection, and a talisman of power.

In one of the scenes of Molière's *Femmes Savantes*, in which some of the ladies had just been indulging in a strain of florid and exaggerated sentiment and euphemisms, a poetaster is introduced, with the recommendation of his knowing Greek. On hearing this, the pseudo-learned ladies exclaim, "Greek! good gracious, Greek! how delightful to know Greek!" "Let me embrace you, sir," exclaims one of them, "for the love of Greek," and her example is followed by two of her friends. A fourth, who looks on things through the medium of common sense, begs to be excused from joining in the *accolades*, as she does not understand Greek. Some of the American women of the present day seem to hold medicine in the same admiration that the French women did Greek in the time of Louis XIV; and as these latter proposed to have a learned academy of their own, so the former claim the right to have a Female Medical College. For ourselves, we are not disposed to recommend either Greek or Medicine as branches of female education; but if required to choose for the sex, we should incline to the Greek, under a belief that they would find the study of the Greek particles less dry than that of the epiphyses of the bones, and a disquisition on the digamma preferable to digitating, if not handling the intestines, and arguing on the pathology of fluor albus and chlorosis, or descanting on the several stages of parturition. The sex has a bright example before it of distinction in Greek literature, in the person of the celebrated Madame Dacier, the translator of Homer, and a most acute and critical defender of the great poet. We are not aware of any eminence yet attained by our Doctresses of Medicine, nor do we see how a medical lady could combine so well as the "Grecian" did her domestic duties with her literary pursuits. Madame Dacier showed alike her modesty and her learning, when she wrote in the album of a German traveller, as an apology for her unwillingness to place herself among his learned friends, a sentence from Sophocles: "Silence is the female's ornament." Shall we ever see this adopted as a motto on the standard of the Women's Rights party? If it should happen that our Doctress becomes a mother,—a very probable event,—would she imitate the wife of the learned Budæus, who gave birth to eleven children, and who, without neglecting their education, aided her husband in comparing passages and transcribing quotations; was ever at his side, and ever assiduous, when not engaged in her domestic duties.

We shall be told that a female practitioner of medicine could not find time to aid her husband in this way, even if he were a literary one,—occupied as she would be with her professional engagements away from home. There are, however, some obligations which she must fulfil, such as those connected with her maternity, and which would seriously interrupt her intercourse with her patients. In the latter period of gestation, when she is seen entering the sick room with an unmistakable abdominal development, very different and less pleasing ideas are suggested to the mind of the patient and his friends, than those that would arise at the sight of a doctor of the Falstaff genus, whose "belly is with good fat capon lined." The first tells of approaching cessation of medical attendance, with consequent discouragement to both parties, and the latter brings with it smiles and pleasant company. Time rolls on, and on a certain morning the doctress mother sends word to her circle of patients that she must be excused from visiting them for the next month, owing to an increase of her family, which took place during the night. With inquiries after her health she receives, as we learn through "a medium," the two following among other missives of the same nature. The first is from a lady, and runs as follows: "MY DEAR DOCTRESS: How could you serve me so? You deceitful thing! I was full of hopes, from what you told me, knowing as you are in such matters, that you would let me get the start of you, so that I might have the benefit of your invaluable services in my hour of trial. I counted, also, on hearing you repeat, during my pains, some of those sweet verses which our strong-minded sister, the poetess, composed to inspire us with true womanly courage on the occasion. If those other ones, by Clio, were set to music, and played on the piano, they would, I am sure, be more soothing to us, and safer than either chloroform or ether, or that other newly-discovered thing, the name of which sounds like Emmeline. What makes it more unfortunate for me, I am afraid that my unenlightened,—I was going to say barbarian,—husband will not give his consent to my being attended, during my confinement, by your interesting protegee, Marcia. To say the truth, this young person fails to give us desired confidence: you must have noticed that she still retains some of the old-fashioned modesty, which used to be quite common before the late happy reform; and that when certain professional matters are talked over, she actually blushes, as if she had never been present at a Woman's Rights Convention, or followed a course of lectures in a Female Medical College. The upshot, I fear, will be that I shall have to call in one of those odious men-midwives,—the very word itself shows what interlopers they are,—who will offer his stale consolations, and perhaps retail an old nursery joke. To do the creature justice, however, I must acknowledge, that in one of my confinements he saved my life by his prompt and decided course, when all around were panic-struck; some of them ready to faint and some to shriek. I hope the dear little love is doing well; and that your husband will feel that it is his duty to take care of it when you are visiting your patients. Believe me to be your ever grateful and attached SELINA."

The second missive is from a hypochondriac gentleman: it is, of course, shorter and more carefully worded than Selina's familiar epistle: "DEAR MADAM: In the interesting situation in which you now find yourself, I must not hope to receive the benefit of your professional visits for some time to come. I hope your nerves do not give you so much trouble as mine do. I suffer more than usual from palpitations this morning. I was not able to eat more than half a rockfish, nor to drink more than a pint of milk for breakfast. What can I take to give me an appetite? If you have no other preference yourself, I would be quite content to be visited by the young doctress (no intimation this, I assure you, that anybody thinks you are old), whom you once brought with you, that she might hear me tell some of my peculiar symptoms, which, you said, were not described in the books. It seems to me that I could enlarge the list of my nervous feelings, and especially palpitations and flushings, with a shade of vertigo, while this young lady is feeling my pulse, and looking into my eyes to ascertain whether they have lost their jaundiced hue. I am almost sure that the musical tones in which she would give her directions for my conduct will go far to raise me from despondency for the rest of the day. With earnest wishes for your early convalescence, I beg leave to subscribe myself, dear madam, your trusting patient, SOLOMON DOUBTFUL."

Gestation over, lactation begins, and the engrossing claims of the infant, very audibly made known, cannot be disregarded. Here is another cause, if not of absolute detention in her house, at least of frequent interruptions to the professional round of the doctress, who must hurry home to suckle the baby, or if she is detained with a case of midwifery, the little body will have to be sent for and get its food in the room of the patient. A question now comes up which we are unable to solve, viz.: whether the lullaby and rocking, for the purpose of soothing the infantile visitor, will have an equally composing effect on the lady who is in labor. There will be some difference of opinion on this point. The crisis reached, and the birth having taken place, it is more than probable that the cries of the new-comer will rouse to a response, on the same key of C sharp, the baby of the doctress; and then will be heard a duet, the notes of which were never scored by Rossini or Verdi, but which may, for a while, make the young mother believe that she has given birth to twins.

Much, under the new order of things, must be expected from compliant husbands; for as there is a circle of family and domestic duties to be discharged, it is quite obvious, that if the wife assumes the work of the man, out of doors, he must, as far as lies in his power, supply her place in the nursery, the kitchen, and the laundry, and display management and administrative talents, never before dreamed of in his philosophy. That he will often be at fault on these occasions need not excite surprise, when a lady herself, who has the direction of affairs, and devotes herself steadily to her household duties, meets with frequent annoyances and embarrassment from the display of the reserved rights of her 'helps.' Hence the lady doctress, if she comes home for a short time at noon, will not unlikely learn, if she has time to listen at all to her husband's narrative, that the nurse threatens to leave, as it was no part of the agreement that she should have the baby in her arms, or be rocking it in the cradle, all the time, without having a minute she could call her own. Besides, it won't sleep, and she will be obliged to give it something to compose it, and to stop those horrid colics which are always worse after it gets spoon victuals. Sally, the chambermaid, declares that she cannot stay if little Johnny is allowed to kick and scratch and bite her so. Wilhelmina, the oldest daughter, instead of going to school, has gone to attend a meeting of a Woman's Rights Society, which she has heard her Ma talk so much about. As to James, who boasts of belonging to Young America, he has taken a holiday, for the purpose of acting the part of amateur supernumerary in a travelling menagerie. But what matters all this? The doctress, self-sacrificing soul, has a great duty to perform: she feels that she has a call to support the dignity and extend the rights of woman; and she is determined to act up to her part, cost what it may, even at the risk of her becoming almost a man!

Some may say, that a female physician need not marry, but continue, as many of her sex do, to lead a life of single blessedness; and as there are bachelor doctors, why not spinster doctresses? It is well known, however, that ladies are slow in placing full confidence in an unmarried male physician, and will there not be a risk of the gentlemen being equally squeamish in the case of an unmarried female one. All are not as communicative in describing the symptoms of their maladies as the hypochondriac, referred to just now. We think we hear an invalid say to his wife, "I agree in all you tell me about the merits of your young friend, Doctress A. She is intelligent, well read, and I dare say, in some respects, skilful; but then, you know, my dear, that we men (you must not laugh at our weakness), do not feel quite at our ease in detailing our symptoms and infirmities to a young lady as we would to one of our own sex, or even to the more mature Doctress B., who can laugh us out of our embarrassment, and elicit all she wants to know, and that is a good deal, while scarcely seeming to ask us any questions. She talks so much like a man about one's body and ailments, and the operation of remedies, and enlightens one so freely respecting certain physiological mysteries, that I forget she is a woman! I told her as much the other day, and she thanked me in most grateful terms for the compliment."

We cannot resist certain misgivings in the matter of medical consultations, under the new order of things, especially where a young doctor and a youthful doctress are brought together. On such occasions it will be difficult for the former to maintain an adverse opinion against the seductive eloquence of the lady, to whose wishes he feels himself almost instinctively bound to defer. It will take him some time to be fully aware of the seasoning or indurating process to which his fair fellow-practitioner has been subjected, before he can treat her as if she were one of his own brethren in petticoats.

